# A Novel Genetic TDP‐43 Pig Model Mimics Multiple Key ALS‐Like Features

**DOI:** 10.1002/mco2.70330

**Published:** 2025-09-14

**Authors:** Chunhui Huang, Xiao Zheng, Jiaxi Wu, Jiawei Li, Yingqi Lin, Yizhi Chen, Caijuan Li, Xichen Song, Wei Wang, Zhaoming Liu, Jianhao Wu, Jiale Gao, Zhuchi Tu, Zaijun Zhang, Liangxue Lai, Shihua Li, Xiao‐Jiang Li, Sen Yan

**Affiliations:** ^1^ Guangdong Provincial Key Laboratory of Non‐human Primate Research The Sixth Affiliated Hospital of Jinan University, Dongguan, China; Guangdong Provincial Key Laboratory of Non‐human Primate Research, Guangdong‐Hong Kong‐Macau Institute of CNS Regeneration, Jinan University Guangzhou China; ^2^ Department of Periodontology and Oral Implantology Stomatological Hospital School of Stomatology Southern Medical University Guangzhou China; ^3^ China‐New Zealand Joint Laboratory on Biomedicine and Health CAS Key Laboratory of Regenerative Biology Guangdong Provincial Key Laboratory of Stem Cell and Regenerative Medicine Guangzhou Institutes of Biomedicine and Health Chinese Academy of Sciences Guangzhou China; ^4^ Institute of New Drug Research College of Pharmacy Jinan University Guangzhou China; ^5^ Department of Neurology Guangzhou Red Cross Hospital Faculty of Medical Science Jinan University Guangzhou China

**Keywords:** amyotrophic lateral sclerosis, APOE4, movement disorder, pig model, SOD1, TDP‐43

## Abstract

Amyotrophic lateral sclerosis (ALS) is a devastating neurodegenerative disease that lacks ideal models to comprehensively recapitulate its pathological features. TDP‐43 pathology, a hallmark of neurodegenerative diseases, plays a critical role in disease progression. Given the anatomical and physiological similarities between pig and human brains, large animal models offer a unique advantage in more accurately simulating patient‐specific disease characteristics. In this study, we rapidly established a TDP‐43‐induced neurodegenerative disease model in pigs through ear vein injection of the TDP‐43^M337V^ virus. Disease progression was systematically evaluated using behavioral assessments and pathological analyses. This porcine model produced extremely severe motor dysfunction accompanied by significant muscle atrophy and fibrosis. Additionally, characteristic TDP‐43 pathological phenotypes were observed, including degeneration of spinal motor neurons and proliferation of glial cells in both the brain and spinal cord. Notably, TDP‐43^M337V^ induction led to a significant upregulation of TMEM106B, SOD1, and APOE4 levels. This TDP‐43 porcine model recapitulates multiple key features of ALS and serves as a valuable complement to existing animal models, providing a robust platform for investigating TDP‐43‐related pathogenic mechanisms of TDP‐43 and developing effective therapeutics.

## Graphical abstract

A scalable ALS pig model was quickly and stably established by injecting TDP‐43(M337V) virus into the ear vein. This model can simulate a variety of disease characteristics of ALS patients, including severe movement disorders, muscle atrophy, motor neuron loss and gliosis. At the same time, many disease molecular features also appeared, such as TDP‐43 pathology, increased SOD1, TMEM106B and APOE4, accompanied by autophagy and lysosomal disorders.

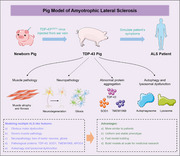

## 1 Introduction

Amyotrophic lateral sclerosis (ALS) is a severe and progressive neurodegenerative disorder characterized by degeneration of both upper and lower motor neurons (MNs). Patients with ALS typically experience muscle weakness and atrophy that advance rapidly to paralysis and fatal outcomes [1]. About 10% of ALS patients demonstrate some form of family history, classifying it as familial ALS (FALS), while the other approximately 90% of cases are considered sporadic ALS (SALS) [1, 2]. Over 30 genetic variants have been strongly linked to ALS pathogenesis, with frequent mutations observed in *superoxide dismutase 1* (*SOD1*), *trans‐reactive DNA‐binding protein* (*TARDBP*, encoding TDP‐43 protein), *chromosome 9 open reading frame 72* (*C9ORF72*), and *fusion sarcoma* (*FUS*) [3]. Notably, TDP‐43 is a ubiquitously expressed DNA/RNA‐binding protein whose mutations are involved in multiple pathogenic mechanisms in ALS progression [4]. Mutations in the *SOD1* gene account for 10%–20% of FALS cases and in up to 7% of SALS, whereas mutations in *TDP‐43* are only a rare cause of ALS [5, 6]. However, the pathological features of TDP‐43 are present in almost all ALS patients, suggesting that altered function of TDP‐43 is an important pathogenic factor [7, 8].

Animal models play a vital role in investigating disease mechanisms and facilitating drug development. Rodent models are commonly employed due to their cost‐effectiveness and well‐established genetic manipulation techniques. While these models exhibit ALS‐like symptoms and have significantly contributed to understanding ALS pathology, they fail to fully replicate all disease features. For example, cytoplasmic TDP‐43 accumulation is a pathological hallmark in the brains of ALS patients [5, 6, 9], which is not prominent in most of mouse models expressing mutant TDP‐43. In contrast, large animal models, such as transgenic TDP‐43 model and viral injection of TDP‐43 monkeys [10, 11], show the accumulation of TDP‐43 in the cytoplasm, indicating that large animal models can more closely mimic the pathophysiological features of ALS than rodents [12]. However, these large animal models present challenges: transgenic pigs show variable genetic profiles due to random transgene integration, along with breeding difficulties, while monkey models involve substantial costs and lengthy experimental timelines. These limitations hinder their widespread adoption and slow progress in ALS research.

Pigs share significant similarities with humans in terms of anatomy, physiology, immune function and genetic makeup, making them highly suitable as translational models for human diseases [13]. Unlike small animals, whose brain structures differ substantially from humans, pigs possess brain characteristics that more closely resemble those of humans, rendering them particularly valuable for modeling neurodegenerative disorders. To capitalize on these advantages, we developed a rapid ALS pig model through ear vein injection of TDP‐43^M337V^ virus. This model successfully recapitulates key ALS features, including pronounced motor impairment and behavioral abnormalities, accompanied by severe muscle atrophy and fibrosis. Furthermore, this porcine model exhibits progressive degeneration of spinal MNs due to the emergence of TDP‐43 pathology, while significant proliferation of glial cells is observed in the brain and spinal cord. Importantly, we detected elevated levels of neurodegeneration‐linked proteins (TMEM106B, SOD1, and APOE4) and impaired lysosomal/autophagic pathways. Collectively, this porcine model faithfully reproduces multiple ALS‐relevant pathological and molecular changes, providing a robust platform for mechanistic studies and therapeutic development.

## 2 Results

### 2.1 TDP‐43 Pigs Exhibited Severe Movement Disorders and Behavioral Deficits

**FIGURE 1:**
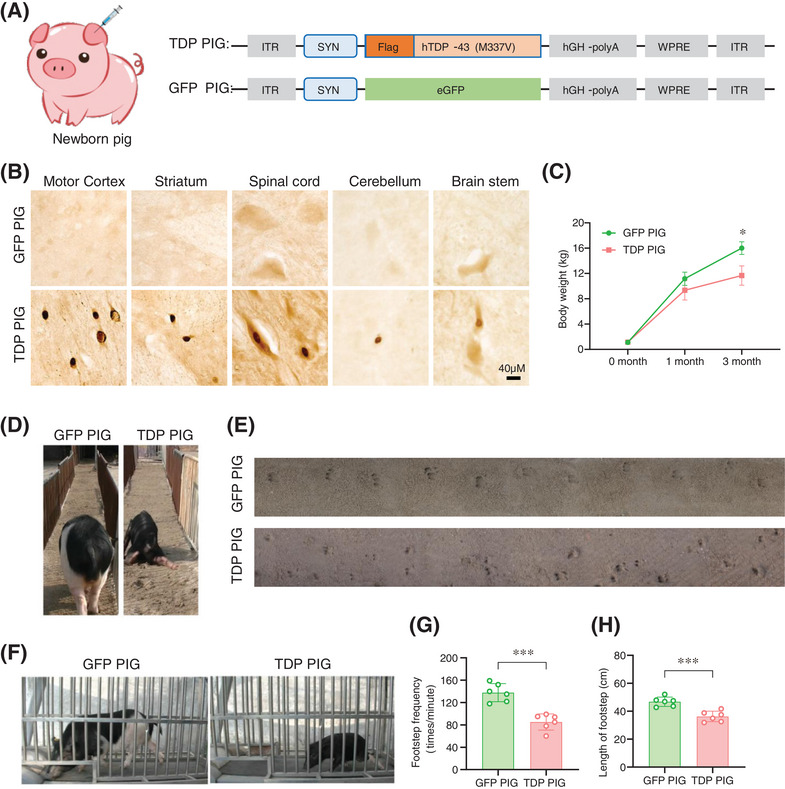
Ear vein injection of AAV9‐SYN‐Flag‐TDP‐43^M337V^ produced severe ALS‐like movement disorders and behavioral deficits in pigs. (A) Schematic diagram of intravenous injection of AAV9‐SYN‐Flag‐TDP‐43 or AAV9‐SYN‐eGFP virus in the ear margin of pigs. (B) AAV9‐SYN‐Flag‐TDP‐43 was successfully expressed in the central nervous system of pigs by ear vein injection. Immunohistochemical staining of Flag tags in the central nervous system. (C) Body weight records for GFP PIG and TDP PIG (*n* = 6 per group, data are mean ± SD, *p < 0.05, two‐way ANOVA Sidak's multiple comparisons test was used for statistical, two‐tailed). (D) TDP‐43 pigs showed mobility impairment and limb incoordination. (E) Representative images of footprint experiments. (F) Representative images of treadmill experiments. (G) Quantification of footstep length in the footprint experiment. The distance between footprints of TDP‐43 pigs was significantly shortened (*n* = 6 per group, data are mean ± SD, ***p < 0.005, unpaired *t* test was used for statistical, two‐tailed). (H) Quantification of stride frequency per minute in treadmill experiment (*n* = 6 per group, data are mean ± SD, ***p < 0.005, unpaired *t* test was used for statistical, two‐tailed).

We have previously demonstrated that AAV viruses were widely distributed in the central nervous system (CNS) and the periphery tissues of minipigs when the ear vein injection of AAV was performed in newborn pigs [14]. Therefore, we injected AAV9‐SYN‐Flag‐TDP‐43^M337V^ and AAV9‐SYN‐eGFP viruses into the ear vein of 1‐week‐old piglets to establish a TDP‐43 pig model (TDP PIG) and control pigs (GFP PIG), respectively (Figure 1A). To verify the expression of exogenous TDP‐43^M337V^, we stained various brain regions. Three months after viral expression, we detected that exogenous TDP‐43 (labeled by Flag) could be expressed in the CNS, including the motor cortex, striatum, spinal cord, cerebellum, and brainstem (Figure 1B), but could not be found in peripheral tissues such as heart, liver, lung, kidney, and muscle (Figure S1A). The body weight of TDP‐43 pigs decreased significantly as the virus was expressed in the CNS (Figure 1C). Importantly, we observed that TDP PIG exhibited movement disorders, limb incoordination, and involuntary falls while walking (Figure 1D, Videos S1 and S2). In the footprint test, TDP PIG had a significantly reduced stride length compared to GFP PIG and dragged its legs on the ground when traversing sandy terrain (Figure 1E,H, Videos S3 and S4). In the treadmill test, we recorded the stride length of the pigs running on the treadmill at a speed of 3.0 km/h for 1 min. The gait frequency of TDP PIG pigs was significantly reduced compared with GFP PIG (Figure 1F,G, Videos S5 and S6).

### 2.2 TDP‐43^M337V^ Induced TDP‐43 Pathology in TDP PIG

**FIGURE 2:**
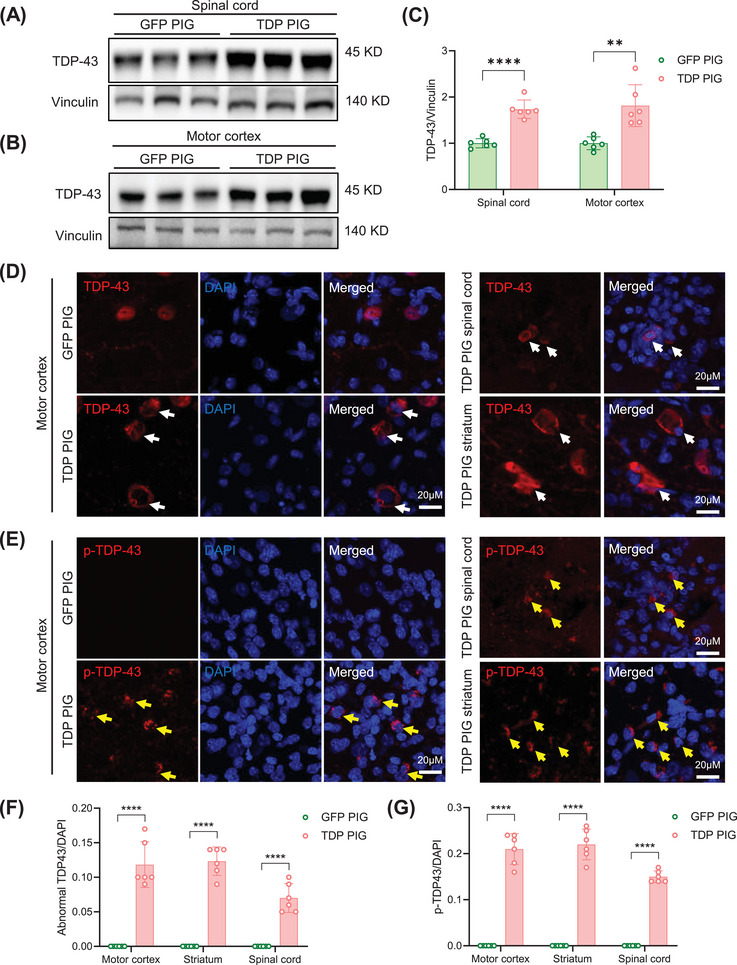
TDP‐43^M337V^ overexpression caused TDP‐43 pathology. (A) Increased expression of TDP‐43 in spinal cord. (B) Increased expression of TDP‐43 in motor cortex. (C) Quantitative statistics of increased TDP‐43 expression in spinal cord and motor cortex (*n* = 6 per group, data are mean ± SD, **p < 0.01, ****p < 0.001, unpaired *t* test was used for statistical, two‐tailed). (D) Representative immunofluorescence of TDP‐43 increase in TDP‐43 porcine cytoplasm. (E) Immunofluorescent staining of p‐TDP‐43 (Ser409/410) in TDP‐43 porcine. (F) Statistical chart of abnormal TDP‐43 amounts in different brain regions (*n* = 6 per group, data are mean ± SD, ****p < 0.001, unpaired *t* test was used for statistical, two‐tailed). (G) Statistical chart of p‐TDP‐43 amounts in different brain regions (*n* = 6 per group, data are mean ± SD, ****p < 0.001, unpaired *t* test was used for statistical, two‐tailed).

Abnormal aggregation of TDP‐43 may lead to damage and death of MNs, which are responsible for controlling muscle movement, thus causing motor dysfunction. To prove whether the severe motor dysfunction of TDP PIG is caused by TDP‐43 pathology, we examined TDP‐43 pathology in the lumbar spine and motor cortex. Western blot results showed that the expression level of TDP‐43 was significantly increased in TDP PIG lumbar spinal cord and motor cortex (Figure 2A–C), while TDP‐43 expression levels in peripheral tissues did not change except muscle (Figure S1C,D, Figure 4E,F). The key change in TDP‐43 toxicity is the accumulation of pathogenic TDP‐43 in the cytoplasm and the formation of cytoplasmic aggregates [7, 8, 15]. Similarly, cytoplasmic accumulation of TDP‐43 was observed in the lumbar spinal cord, motor cortex, and striatum of TDP PIG (Figure 2D,F). Another important TDP‐43 pathology is the aggregation of phosphorylated TDP‐43 [16], and consistent with this, phosphorylated TDP‐43 (Ser409/410) was increased in TDP PIG and formed aggregates (Figure 2E,G).

### 2.3 TDP‐43^M337V^ Induces Pathological Features of Motor Neuron Loss and Gliosis

**FIGURE 3:**
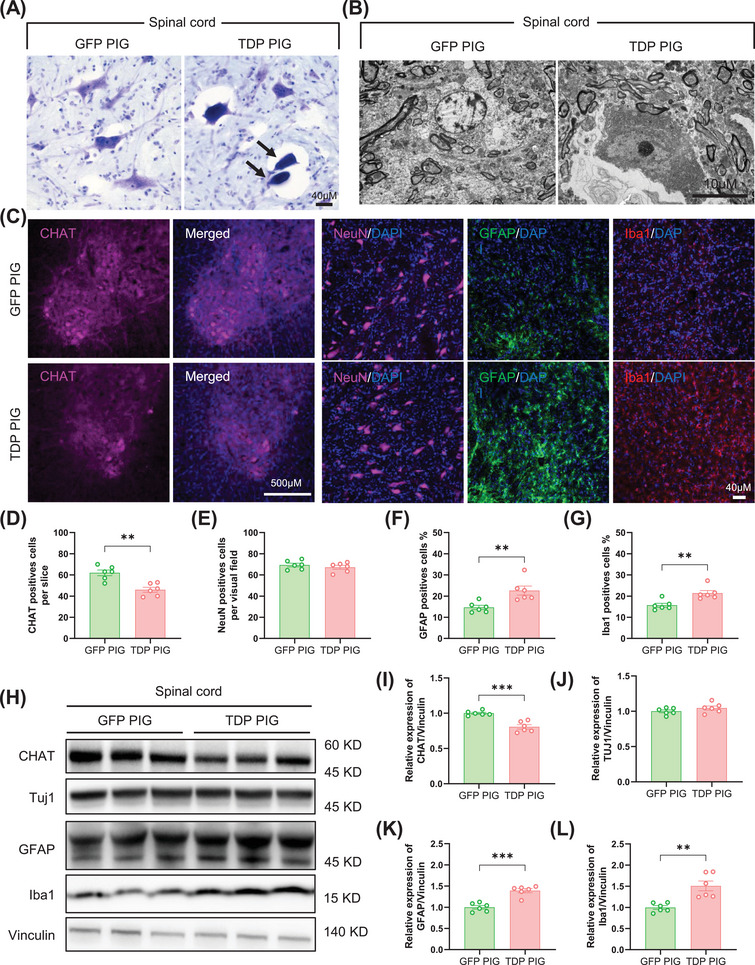
TDP‐43 pigs develop motor neuron degeneration and gliosis in spinal cord. (A) Representative image of toluidine blue‐stained of lumbar spinal cord ventral horn showing degenerating motor neuron with darkened and shrunken cytoplasm in TDP pigs. (B) Transmission electron microscopy image of a degenerating MN with nuclear cavities and electron dense granules in TDP pigs. (C) Representative immunofluorescent staining of CHAT‐positive, NeuN‐positive, GFAP‐positive, and Iba1‐positive cells in the lumbar spinal cord. (D–G) Quantitative statistics of CHAT‐positive (D), NeuN‐positive (E), GFAP‐positive (F), and Iba1‐positive (G) cells in the lumbar spinal cord (*n* = 6 per group, data are mean ± SD, **p < 0.01, unpaired *t* test was used for statistical, two‐tailed). (H) Western blot of CHAT, Tuj1, GFAP, and Iba1 in lumbar spinal cord. (I–L) Quantitative statistics of CHAT (I), Tuj1 (J), GFAP (K), and Iba1(L) in the lumbar spinal cord (*n* = 6 per group, data are mean ± SD, **p < 0.01, ***p < 0.005, unpaired *t* test was used for statistical, two‐tailed).

TDP‐43 pathology exacerbates ALS disease progression by leading to progressive degeneration of MNs. We have found that TDP‐43 pathology is present in the spinal cord and motor cortex of TDP PIG, and then we further tested whether these pathologies cause the degeneration of MNs. We examined the condition of spinal cord MNs using toluidine blue‐staining, transmission electron microscopy (TEM), and immunofluorescence staining. Several degenerated condensed MNs were observed in TDP PIG, which exhibited dark and shrunken cytoplasm with shrinkage of the cell membrane, consistent with degeneration (Figure 3A). TEM images also showed distinct nuclear changes in MNs, characterized by the presence of nucleolar cavities and electron‐dense granules in TDP PIG pigs relative to GFP PIG pigs (Figure 3B). In addition, immunofluorescent staining revealed a significant reduction in CHAT‐positive MNs in the lumbar spinal cord (Figure 3C,D). Consistently, western blot results also showed a significant decrease in CHAT protein levels in TDP PIG spinal cords (Figure 3H,I). Although we did not find a decrease in NeuN‐positive neurons, glial cells were significantly activated in the spinal cord and motor cortex both in immunofluorescence staining and western blot (Figure 3C,E–L and Figure S2). Specifically, in the spinal cord and motor cortex, the number of astrocytes and microglia was significantly increased in TDP PIG as compared to GFP PIG, representing a possible increase in neuroinflammation in TDP PIGs.

### 2.4 Severe Skeletal Muscle Pathology Observed in TDP PIG

**FIGURE 4:**
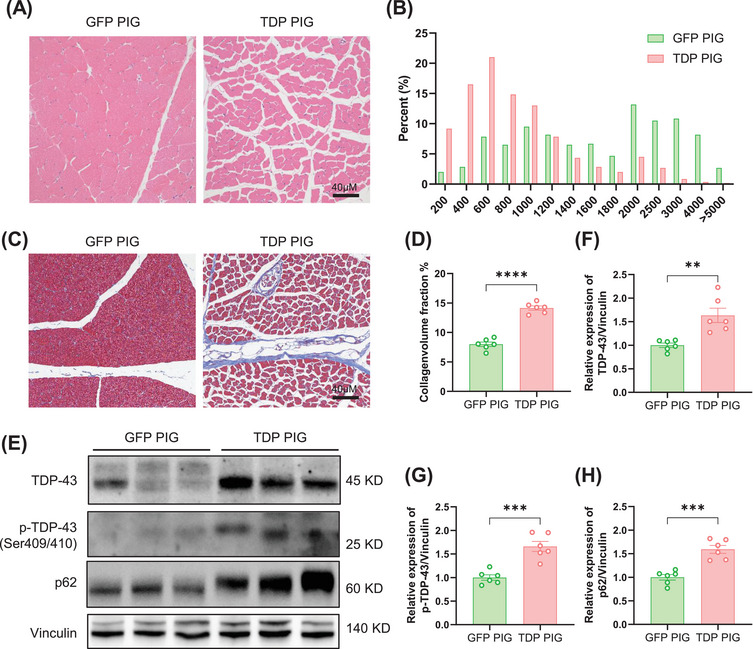
TDP‐43 pigs exhibit severe muscle pathology. (A) Representative H&E staining of skeletal muscle showing muscle atrophy. (B) More than three random fields (corresponding to 100 cross‐sectioned fibers per field) were examined for quantification of muscle fibers of different sizes. (C) Representative Masson staining of skeletal muscle graph showing increased muscle fibrosis in TDP‐43 pigs. (D) Quantitative statistics of skeletal muscle fibrosis (*n* = 6 per group, data are mean ± SD, ****p < 0.001, unpaired *t* test was used for statistical, two‐tailed). (E) The western blot of TDP‐43, p‐TDP‐43 (Ser409/410), and p62 expression levels in skeletal muscle. (F–H) Immunohistochemical staining of TDP‐43 (F), p‐TDP‐43 (Ser409/410) (G), and p62 (H) in skeletal muscle (*n* = 6 per group, data are mean ± SD, **p < 0.01, ***p < 0.005, unpaired *t* test was used for statistical, two‐tailed).

In ALS, muscle pathology is an important aspect of disease progression. As MNs degenerate, the muscles innervated by these neurons undergo a series of changes, such as multiple studies in ALS patients and animal models showing that muscle atrophy is a major feature of ALS [17]. Through HE staining of porcine skeletal muscle, we found that TDP PIG skeletal muscle showed severe atrophy compared with GFP PIG, specifically, muscle fibers gradually became thinner, resulting in a decrease in muscle volume (Figure 4A,B). In neurogenic muscle atrophy, small keratinization may appear on the edges of muscle fibers, and a similar phenomenon occurred in TDP PIG. In addition, Masson staining showed that the proportion of collagen fibers in TDP PIG in ALS was significantly increased compared with GFP PIG, indicating an increase in muscle fibrosis (Figure 4C,D). In ALS, TDP‐43 undergoes abnormal phosphorylation and translocation from the nucleus to the cytoplasm, forming aggregates that can also be detected in muscle tissue [18]. Although transgenic Flag‐TDP‐43 expression was not observed in muscle, significant increases in endogenous TDP‐43, p‐TDP‐43, and p62 were observed, which are common pathological markers in ALS muscles (Figure 4E–H).

### 2.5 TDP PIG Displayed TMEM106B Pathology and Autophagy‐Lysosome Dysfunction

**FIGURE 5:**
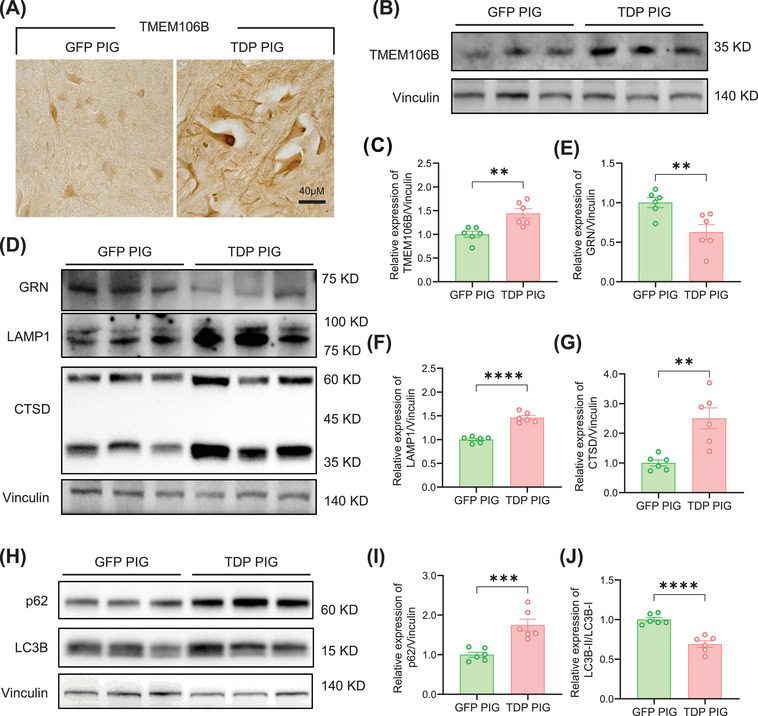
Lysosomal and autophagic dysfunction in TDP‐43 porcine spinal cord. (A) Representative immunohistochemical staining of TMEM106B in spinal cord. (B) Western blot of TMEM106B increase in spinal cord. (C) Quantification of TMEM106B increase in spinal cord (*n* = 6 per group, data are mean ± SD, **p < 0.01, unpaired *t* test was used for statistical, two‐tailed). (D) Western blot of increased GRN, LAMP1, and CTSD in the spinal cord. (E–G) Quantification of GRN (E), LAMP1 (F), and CTSD (G) increases in the spinal cord (*n* = 6 per group, data are mean ± SD, **p < 0.01, ****p < 0.001, unpaired *t* test was used for statistical, two‐tailed). (H) Western blot of increased autophagy marker p62 and the decrease of LC3BII/I in the spinal cord. (I and J) Increased autophagy marker p62 (J) and the decrease of LC3BII/I (J) in the spinal cord (*n* = 6 per group, data are mean ± SD, ***p < 0.005, ****p < 0.001, unpaired *t* test was used for statistical, two‐tailed).

In the brains of ALS patients, the expression level of Lysosomal type II transmembrane protein 106B (TMEM106B) may be correlated with the severity of TDP‐43 pathology. The dysfunction of TMEM106B may lead to the obstruction of the clearance of TDP‐43 in cells, thereby promoting the abnormal aggregation of TDP‐43. This aggregation may lead to neuronal dysfunction and death, thus causing movement disorders in ALS. TMEM106B regulates many aspects of lysosomal function, including lysosomal pH, lysosomal motility, and lysosomal exocytosis. Both increased and decreased TMEM106B levels lead to lysosomal abnormalities [19]. TMEM106B has been identified as a risk gene for ALS, and changes in its levels are critical for ALS neuropathology. It has been reported that TMEM106B directly modifies the development of TDP‐43 pathology in ALS patient brains and ALS animal models [20]. Interestingly, we found that the expression level of TMEM106B was significantly higher in the spinal cord and motor cortex of TDP PIG than that of GFP PIG (Figure 5A–C and Figure S3A–C). Moreover, TMEM106B aggregates were also seen in the cytoplasm in the TDP PIG spinal cord and motor cortex.

Progranulin (GRN) is a secreted glycoprotein localized to the lysosomes and is essential for the normal function of the lysosomes. Heterozygous GRN mutation carriers can develop TDP‐43 pathology in FTLD and show signs of lysosomal dysfunction in the brain, with increased accumulation of lysosomal proteins and lipofuscin [21]. Additionally, the TMEM106B transgenic mouse model was characterized by exaggerated lysosomal abnormalities and increased accumulation of lipofuscin, whereas TMEM106B deletion ameliorated lysosomal dysfunction [22, 23, 24]. Likewise, the expression level of GRN was significantly decreased in the spinal cord of TDP PIG, and the expression of LAMP1 and mature cathepsin D (CTSD) was significantly increased (Figure 5D–G), demonstrating the existence of lysosomal dysfunction. In addition, we also found a significant increase in p62 levels and a significant downregulation of LC3BII/I in TDP PIG, indicating that autophagic flux was reduced and the autophagic process might be inhibited (Figure 5H–J). Similar to the spinal cord, the expression levels of LAMP1, CTSD, and p62 increased in the motor cortex, while the level of LC3BII/I decreased (Figure S3D–I).

### 2.6 TDP PIG Showed an Increase in SOD1 and APOE4 Levels

**FIGURE 6:**
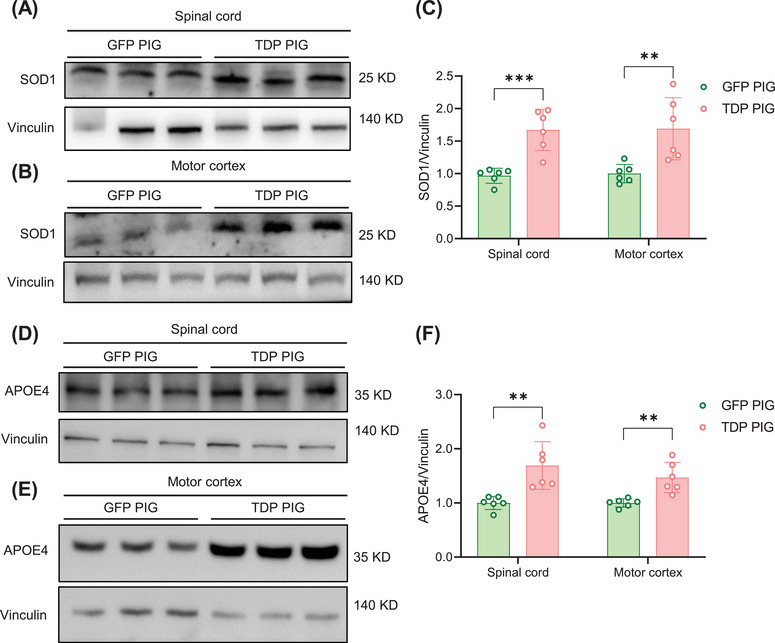
Increased expression of SOD1 and APOE4 in spinal cord and motor cortex of TDP‐43 pigs. (A) Western blot of SOD1 increase in the spinal cord. (B) Western blot of SOD1 increase in motor cortex. (C) Quantification of SOD1 increase in spinal cord and motor cortex (*n* = 6 per group, data are mean ± SD, **p < 0.01, ***p < 0.005, unpaired *t* test was used for statistical, two‐tailed). (D) Western blot of APOE4 increase in the spinal cord. (E) Western blot of APOE4 increase in motor cortex. (F) Quantification of APOE4 increase in spinal cord and motor cortex (*n* = 6 per group, data are mean ± SD, **p < 0.01, unpaired *t* test was used for statistical, two‐tailed).

Currently, no existing model completely replicates the full spectrum of pathological changes seen in ALS patients. Developing a model that can mimic multiple ALS characteristics is crucial for advancing research. In this context, we investigated whether our TDP PIG model exhibits additional ALS‐related pathological markers. SOD1, a key antioxidant enzyme and the first identified major genetic risk factor for ALS, plays a significant role in disease pathogenesis. Notably, recent research has demonstrated that the co‐aggregation of SOD1, TDP‐43, and P62 in spinal MNs contributes substantially to neurodegeneration in ALS [25]. Intriguingly, our findings revealed elevated SOD1 levels in both the spinal cord and brain tissues of TDP PIGs (Figure 6A–C), demonstrating that this model successfully captures this additional pathological hallmark of ALS.

Apolipoprotein E (APOE) is a glycoprotein involved in lipid transport, expressed both in the CNS and peripheral tissues [26]. Emerging evidence links the APOE4 isoform to TDP‐43 pathology. For instance, a Mayo Clinic Brain Bank study found that Alzheimer's disease (AD) cases with comorbid TDP‐43 pathology were more likely to carry the APOE4 allele compared to TDP‐43‐negative AD cases [27]. Another study based on the ROSMAP cohort reported that stage and burden of TDP‐43 pathology was positively associated with the number of APOE4 alleles, even after controlling for amyloid, tau, and Lewy body pathology [28]. Together, these findings suggest that APOE4 may exacerbate TDP‐43 accumulation. However, no existing animal model has directly demonstrated a mechanistic connection between TDP‐43 and APOE4. Interestingly, in our TDP‐43^M337V^ overexpressing pig model (TDP PIG), we observed elevated APOE4 levels in the spinal cord and motor cortex, providing the first in vivo evidence of this association (Figure 6D–F).

### 2.7 RNA Seq Revealed Similar Transcriptome Changes Between TDP PIG and ALS Patients

**FIGURE 7:**
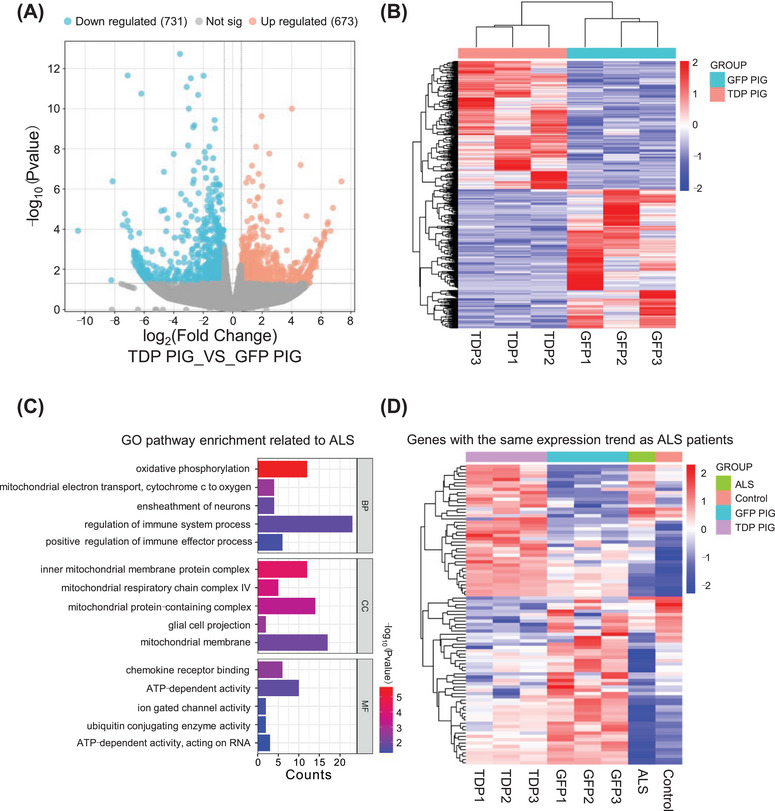
TDP PIG and GFP PIG spinal cord RNA seq results. (A) Volcano plot of differentially expressed genes between TDP PIG and GFP PIG (*n*  =  3 pigs per group). (B) Heat map of 1404 differentially expressed genes between TDP PIG and GFP PIG. (C) GO enrichment analysis of differentially expressed genes between TDP PIG and GFP PIG. (D) Heat map of genes that are consistent with changes in ALS patient data for differentially expressed genes between TDP PIG and GFP PIG.

To explore whether TDP‐43 pig has similar transcriptional changes as ALS patients, we obtained spinal cord tissues of GFP PIG and TDP PIG for RNA seq and compared them with ALS patient sequencing data. The results showed that compared with GFP PIG, TDP PIG had 1404 differentially expressed genes, including 731 downregulated genes and 673 upregulated genes (Figure 7A,B). GO enrichment analysis of these highly variable genes revealed that ALS‐related pathways such as oxidative phosphorylation, ATP activity, mitochondrial structure and function‐related pathways, immune response, and ubiquitination‐related pathways were altered (Figure 7C), which have been reported in ALS patient data [29, 30, 31, 32]. We then obtained the transcriptome data of ALS patients from the DiSignAtlas (http://www.inbirg.com/disignatlas/) database and extracted differentially expressed genes. By comparison, we found that 91 differentially expressed genes in TDP PIG and GFP PIG had consistent changes with the data of ALS patients (Figure 7D). Considering that pig genes have not been fully annotated, some genes cannot be matched with human genes, which means that the actual matched differentially expressed genes should be much larger than 91. Therefore, there are significant differences in gene expression profiles between TDP PIG and GFP PIG, and there are overlapping gene changes with ALS patients, indicating that TDP PIG can be used as a reliable large animal model of ALS.

## 3 Discussion

In this study, we established a porcine model of ALS through ear vein injection of TDP‐43^M337V^ expressing viral vectors. This model successfully recapitulates multiple hallmark pathological features of ALS, including a distinct dyskinesia phenotype, global TDP‐43 pathology, MN degeneration, gliosis, severe muscle atrophy with fibrosis, TMEM106B accumulation, and increased SOD1 and APOE4 levels. While no existing model completely reproduces all aspects of ALS pathology, this porcine system provides a unique platform to investigate the pathogenic mechanisms by which mutant TDP‐43 drives diverse neurodegenerative processes, and the interplay between these pathological events in ALS progression.

The application of porcine models is playing an increasingly important role in the field of biomedicine. The TDP‐43 pig model we established is based on large animals. Compared with rodents, pigs are closer to humans, especially in the CNS [33], allowing for investigating the pathological features and phenotypes that may not occur in rodents [12]. Current evidence suggests that most transgenic rodent models lack overt neurodegeneration, which poses challenges and barriers to using them to rigorously test the efficacy of therapeutics against neurodegeneration. Recent studies using large animals (pigs and monkeys) have revealed important pathological events that resemble neurodegeneration in patient brains but cannot be produced in small animal models [12]. Especially, most of genetically modified rodent models, including mutant SOD1 transgenic mice, *TDP‐43* transgenic mice, *C9orf72* transgenic mice, *FUS* transgenic mice, fail to recapitulate the typical and overt neurodegeneration seen in patients. In contrast, in non‐human primates, our group found that TDP‐43 (M337V) induces SQSTM1 mRNA instability by binding to a unique 3′UTR sequence in non‐human primate SQSTM1 transcripts, thereby selectively reducing SQSTM1 expression in monkey brain compared with mouse brain [34]. In addition, primate‐specific caspase‐4 can remove the NLS‐containing N‐terminal domain of TDP‐43 and generate fragmented TDP‐43 that accumulates in the cytoplasm [10]. Importantly, we previously found in our TDP‐43 transgenic pigs the same cytoplasmic localization of PSF and NeuN and abnormal PSF‐related neuronal RNA splicing as in the brains of ALS patients [35]. Therefore, it is important to emphasize the uniqueness of large animals in modeling neurodegenerative diseases.

Moreover, most ALS‐related mouse models only target the corresponding FALS, but are powerless against most SALS pathologies. In contrast to the rodent models, large animal models such as TDP‐43^M337V^ or SOD1 transgenic pigs and virus‐injected monkey models can display this important phenomenon [10, 35, 36, 37]. Clinical sample testing of ALS patients has identified multiple pathological features of ALS, such as TMEM106B pathology and SOD1 pathology, as well as the direct link between APOE and ALS [20, 25, 28]. However, there is currently no animal model that can fully simulate the pathology of ALS patients. Our results show that TDP PIG exhibits TMEM106B pathology, with high expression levels of SOD1 and APOE, outlining multiple ALS features. It is worth mentioning that our TDP‐43 pig model cannot only recapitulate the neurodegenerative changes and the accumulation of TDP‐43 in the cytoplasm but also mimic the multiple pathological characteristics of ALS, which is closer to the characteristics of SALS and FALS, thus imitating most ALS cases.

Large animal models better replicate the progressive neuropathology observed in ALS patients. Currently available ALS large animal models include TDP‐43^M337V^ transgenic pig, SOD1 transgenic pig, and TDP‐43^M337V^‐injected monkey models [10, 11, 35, 36, 37]. Different from the previously established transgenic pig models, the TDP‐43 pig model we presented can be rapidly and easily established via vein injection of viral vectors in the newborn wild‐type pigs. An advantage of virus‐mediated porcine models of ALS over transgenic porcine models created to date is that they can be generated in large enough numbers to appropriately drive research into novel biomarkers of disease progression and promising treatments. Moreover, our TDP‐43 pigs avoid random germline integration, eliminating variability from copy number and insertion site effects seen in transgenic animals. In the transgenic TDP‐43 pig model, both NeuN‐positive and CHAT‐positive neurons were significantly lost at 10 months [35]. In the intravenous injection of TDP‐43 PIG model, NeuN‐positive neurons had not yet degenerated at 3 months, and only CHAT‐positive neurons degenerated. This is limited by the dose and expression time of TDP‐43 (M337V) virus, and more studies are needed to confirm. Importantly, in the early stages of ALS, CHAT‐positive MNs may be affected first, showing functional abnormalities and reduced numbers [38, 39]. As the disease progresses, NeuN‐positive neurons are gradually lost, which may include neurons in the cerebral cortex, brainstem, and spinal cord. This model thus captures the initial phase of ALS, where MN dysfunction precedes broader neuronal loss, providing a platform to study disease onset and progression.

Compared with the non‐primate models, pigs offer the advantage of rapid large‐scale reproduction, enabling researchers to obtain sufficient numbers for comprehensive ALS pathology studies and therapeutic development. While direct brain or spinal cord injections can induce localized neurodegeneration and TDP‐43 pathology restricted to injection sites (often without muscle involvement), our intravenous viral delivery model achieves widespread CNS expression of mutant TDP‐43. This systemic approach recapitulates key ALS features including muscle atrophy and fibrosis, making it a more clinically relevant model of the disease.

Large animal models provide critical advantages for investigating disease mechanisms and developing therapies for neurodegenerative disorders [12, 40, 41]. Currently, only two FDA‐approved drugs are available to treat ALS patients, both with modest efficacy and high cost. Over the past decade, at least 18 drugs have been tested in phase II or III clinical trials for ALS, but all have failed [42]. Animal models are crucial for the translational studies of drugs. These repeated failures highlight the urgent need for improved preclinical models that can better predict therapeutic outcomes. The porcine model is the most promising model for translational biomedical research [43]. Our established pig model successfully recapitulates multiple key ALS pathological features, making it especially valuable for evaluating potential treatments and advancing drug development efforts.

Collectively, we anticipate that our intravenous TDP‐43 pig model will serve as an important new resource for the ALS research field, facilitating both the identification of novel disease progression markers and the assessment of potential therapeutic interventions.

## 4 Materials and Methods

### 4.1 Animals and Ethics Statement

Study Approval: Animal use and care followed the NIH Guide for the Care and Use of Laboratory Animals. All procedures involving animal use and care were conducted in strict compliance with the NIH Guide for the Care and Use of Laboratory Animals. The experimental protocol was reviewed and approved by the Institutional Animal Care and Use Committee (IACUC) at the Guangzhou Institute of Biomedicine and Health (GIBH), Chinese Academy of Sciences. This study was conducted in strict accordance with the “Guide for the Care and Use of Laboratory Animals (2011)” to ensure both personnel safety and the welfare of the animals involved.

Bama pigs, a local breed originating from Southern China, were raised at the GIBH, Chinese Academy of Sciences (Animal Welfare Assurance #N2019083). The experiments utilized wild‐type bama pigs, which were housed indoors at room temperature in the Animal Center of Guangzhou Institutes of Biomedicine and Health, with free access to standard food and water. Three‐year‐old wild‐type sows were mated with boars, and piglets were selected from their offspring and randomly divided into two groups (GFP PIG and TDP PIG), with six pigs (half male and half female) in each group for subsequent experiments.

### 4.2 Virus Production and Injection

The virus expressing TDP‐43^M337V^ is derived from pUAS‐hTDP‐43^M337V^ plasmid [44], which encodes human mutant TDP‐43^M337V^. The vector was packaged by PackGene Biotech with the AAV9 serotype. Purified viruses were stored at −80°C. The genomic titer of the purified viruses (vg) (approximate 10^13^ vg/mL) was determined by PCR method. The same litter of 1‐week‐old Bama pigs were injected with AAV9‐SYN‐Flag‐TDP‐43^M337V^ or AAV9‐SYN‐eGFP as the experimental group or the control group. The virus injection method was as previously described [14]. The pigs were anesthetized using 1.5% isoflurane, and the injection site was prepared by disinfecting with 10% povidone‐iodine (betadine solution) followed by 75% ethanol. Using a 30G needle connected to a 1‐mL Hamilton syringe, we administered the viral solution (100 µL of 10^13^ vg/ml diluted in 1‐mL saline) via slow auricular vein injection over a 5‐min period. Following infusion, the needle remained in situ for an additional 3 min before careful withdrawal. Postoperative care included maintaining the pigs on a warming pad during anesthesia recovery, after which the awakened piglets were returned to their mothers.

### 4.3 Postmortem Examination and Specimen Harvesting

Three months post‐viral injection, pigs were humanely euthanized through deep anesthesia induced by intraperitoneal administration of 3–5 mL atropine, followed by 10–12 mg/kg ketamine. Transcardial perfusion was then performed using 3 L of sterile 0.9% NaCl solution via the left ventricular chamber. Following extraction from the cranial vault, the brain was bisected sagittally: the left hemisphere was rapidly frozen on dry ice after regional dissection, while the right hemisphere was immersion‐fixed in 10 volumes of 4% paraformaldehyde (PFA) for 48 h.

### 4.4 Behavioural Analysis

The motor activity of the pigs was examined as described in our previous study [40]. For the treadmill test, the pigs were placed on a treadmill with a closed cage to assess their running ability. The closed cage allowed the pig to run on the conveyor belt. The pigs were subjected to treadmill training for 3 consecutive days before testing. The speed of the treadmill was kept at 1.5 km/h for 1 min. The treadmill running test was performed three months after virus injection. And the footstep frequency of each pig was counted within one minute.

For the gait test, we used a footprint tracking method described in the previous study [40]. Pigs were trained to pass through the sandy pathway (80 cm wide and 4.5 m long), and their footprints during the movement process were recorded by a camera. The stride lengths for front and rear footprints on the pictures were measured.

### 4.5 Western Blot Analysis, Immunohistochemistry, Immunofluorescence, and Transmission Electron Microscope

The following antibodies were used: Flag (1:1000, F1804), Flag (1:1000, MA1‐91878), TDP‐43 (1:1000, 10782‐2‐AP), TDP‐43 (G400, 1:1000, CST3448), p‐TDP‐43 (Ser403/404) (1:500, 66079‐1‐Ig), NeuN (1:1000, ab177487), GFAP (1:1000, MAB360), Iba1 (1:500, 019–19741), Iba1 (1:1000, ab178846), CHAT (1:500, AB143), Tuj1 (1:1000, MAB1195), SQSTM1/p62 (1:1000, ab91526), TMEM106B (1:1000, 20995‐1‐AP), progranulin (GRN) (1:1000, 10053‐1‐AP), LAMP1 (1:1000, ab62562), CTSD (1:1000, CST2284), LC3B (1:1000, CST3868), SOD1 (1:1000, H00006647‐D01P), α‐tublin (1:1000, MA1‐80017), vinculin (1:1000, 66305‐1‐Ig), and GAPDH (1:1,000, 60004‐1‐Ig). Secondary antibodies were all from Jackson ImmunoResearch Laboratories and Abcam.

For western blotting, pig tissues were ground using a Luka grinding instrument and lysed in ice‐cold RIPA buffer (50 mM Tris pH 8.0, 150 mM NaCl, 1 mM EDTA pH 8.0, 1 mM EGTA pH 8.0, 0.1% SDS, 0.5% DOC and 1% Triton X‐100) containing Halt Protease Inhibitor cocktail (Thermo Fisher) and phenylmethylsulfonyl fluoride (1 mM). The tissue lysates were incubated on ice for 30 min and centrifuged at 10,000 r.p.m. for 10 min. Equal amounts of proteins were loaded onto an SDS‐PAGE system, transferred to a PVDF membrane, and the blots were blocked with 5% fat‐free milk/PBS for 1 h at room temperature and then incubated with primary antibodies diluted in 3% BSA/tris‐buffered saline with 0.1% tween‐20 detergent (TBST) overnight at 4 °C. The membranes were then incubated with HRP‐conjugated secondary antibodies in 5% milk/TBST for 1 h at room temperature. After 3 washes in TBST, ECL Prime (GE Healthcare) was used to detect immunoreactive signals on the blots.

For immunohistochemistry, the isolated pig brain tissues were fixed for 48 h in 4% PFA/0.01 M phosphate buffer and then transferred into 30% sucrose to dehydrate at 4 °C. Coronal sections (30 µm) of porcine brain and lumbar spinal cord were cryosectioned using a freezing microtome. Sections were mounted on slides and fixed with 4% PFA in 0.01 M phosphate buffer (10 min), followed by blocking with 4% normal goat serum containing 0.1% Triton X‐100 in PBS (30 min). Primary antibody incubation was performed overnight at 4°C in 3% BSA/2% normal goat serum/TBST. For DAB staining, Avidin‐Biotin Complex kit (Vector ABC Elite) was used. For immunofluorescence analysis, DAPI (1:1000) was added with secondary antibodies. Microscopic images were acquired using a Zeiss LSM 800 confocal microscope, Zeiss Axio Imager A2 inverted scope, or a TissueFAXS PLUS tissue analysis system (TissueGnostics). TEM study of pig spinal cord was performed by Servicebio (Wuhan, China).

### 4.6 HE and Masson Staining

Pig skeletal muscle was immersion‐fixed in muscle fixative solution (Bouin's fluid, 15990‐01) for 48 h, cryoprotected in 30% sucrose, and sectioned at 5 µm thickness. HE staining and Masson staining were performed by Servicebio (Wuhan, China). Microscopic images were acquired using TissueFAXS PLUS tissue analysis system (TissueGnostics).

### 4.7 RNA Seq

Total RNA was extracted from the lumbar spinal cord of wild‐type pigs injected with either AAV9‐SYN‐Flag‐TDP‐43^M337V^ or AAV9‐SYN‐eGFP via the auricular vein using RNAiso Plus (TaKaRa). For each sample (*n* = 3 per group), 2 mg of RNA was submitted to HeQin Biotechnology Corporation (Guangzhou) for RNA sequencing and database construction. Library preparation was performed with the NEBNext Ultra RNA Library Prep Kit for Illumina (E7530L, NEB) following the manufacturer's protocol. Sequencing was conducted on the Illumina platform, generating 150‐bp paired‐end reads. Raw sequencing data were quality‐filtered using Trimmomatic to remove adapters and low‐quality reads [45]. Clean reads were aligned to the pig genome (Ensembl) using STAR [46], producing sam files. These were converted to sorted and indexed BAM files using SAMtools [47]. Transcript abundance was quantified with StringTie, and a read count matrix was generated using the “prepDE.py” script [48]. Differentially expressed genes (DEGs) were identified using DESeq2 with thresholds of |log2FC| > 0.585 and *p*‐value < 0.05 [49]. Gene Ontology (GO) enrichment analysis was performed in Hiplot Pro (https://hiplot.com.cn/), a comprehensive web service for biomedical data analysis and visualization, retaining terms with *p* < 0.05. Principal component analysis (PCA) and heatmap visualization were conducted using the R package carrieUBC.

### 4.8 Statistical Analysis

When every two groups were compared, statistical significance was assessed with the two‐tailed Student's *t*‐test. Data are presented as mean ± SD. For pathological examination, western blotting, and RNA‐seq, at least three animals per group were used. Calculations were performed with GraphPad Prism software (GraphPad Software). A *p*‐value of 0.05 was considered statistically significant.

## Author Contributions

C. H. conducted the experiments, and performed analysis and writing. X. Z. conducted the experiments and performed the analysis. J. W., J. L., Y. L., Y. C., C. L., and X. S. conducted the experiments. W. W., Z. L., J. W., J. G., and Z. T. collected data. Z. Z., L. L., S. L., and X.J. L. supervised the project. S.Y. design and supervised the project, and performed writing and editing of final version of the manuscript. All authors have read and approved the final manuscript.

## Ethics Statement

Bama pigs, indigenous to Southern China, were bred at the animal facility of the Guangzhou Institute of Biomedicine and Health (GIBH), Chinese Academy of Sciences (Animal Welfare Assurance #N2019083). All procedures involving animal use and care were conducted in strict compliance with the NIH Guide for the Care and Use of Laboratory Animals. The experimental protocol was reviewed and approved by the Institutional Animal Care and Use Committee (IACUC) at the Guangzhou Institute of Biomedicine and Health (GIBH), Chinese Academy of Sciences.

## Conflicts of Interest

The authors declare no conflicts of interest.

## Supporting information



Figure S1.tif

Figure S2.tif

Figure S3.tif

Movie S1.mp4: TDP PIG fell involuntarily while walking.

Movie S2.mp4: TDP PIG exhibited movement disorders, limb incoordination.

Movie S3: Recorded video of TDP PIG conducting sand experiments.

Movie S4: Recorded video of GFP PIG conducting sand experiments.

Movie S5: Recorded video of TDP PIG conducting treadmill experiments.

Movie S6: Recorded video of GFP PIG conducting treadmill experiments.

## Data Availability

The raw data used and/or analyzed during the current study are available from the corresponding author on reasonable request. The RNA‐seq data are publicly available in the NCBI GEO database under accession number PRJNA1255726 and in the CNCB‐NGDC under accession number PRJCA041971.
