# Supplementary figures and images for: A Novel Genetic TDP‐43 Pig Model Mimics Multiple Key ALS‐Like Features
Source: MedComm (2020). 2025 Sep 14;6(9):e70330. doi: 10.1002/mco2.70330 (PMC12434190; doi:10.1002/mco2.70330)

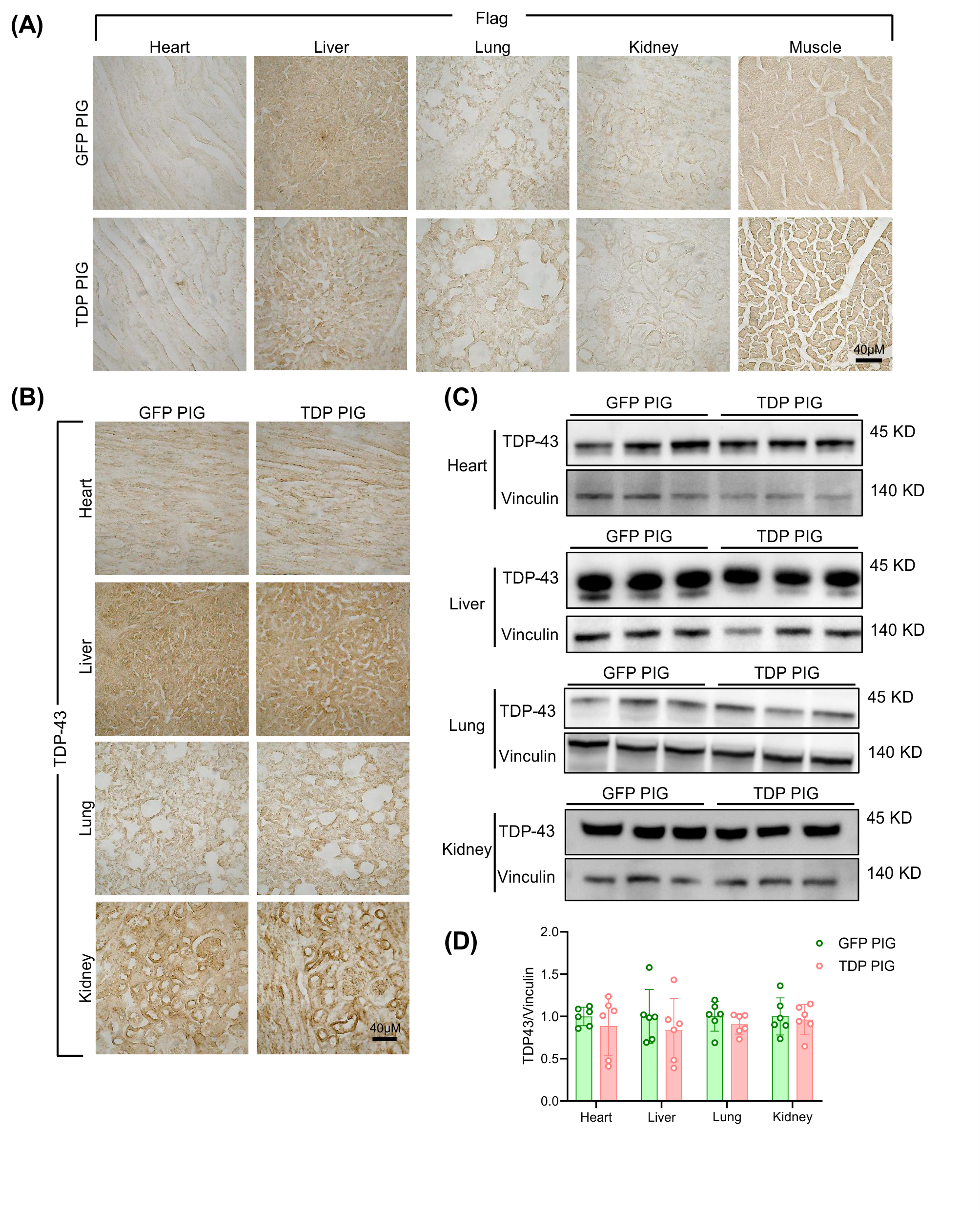

Supplement: Supplementary file 1 — Figure S1.tif [file MCO2-6-e70330-s009.tif]

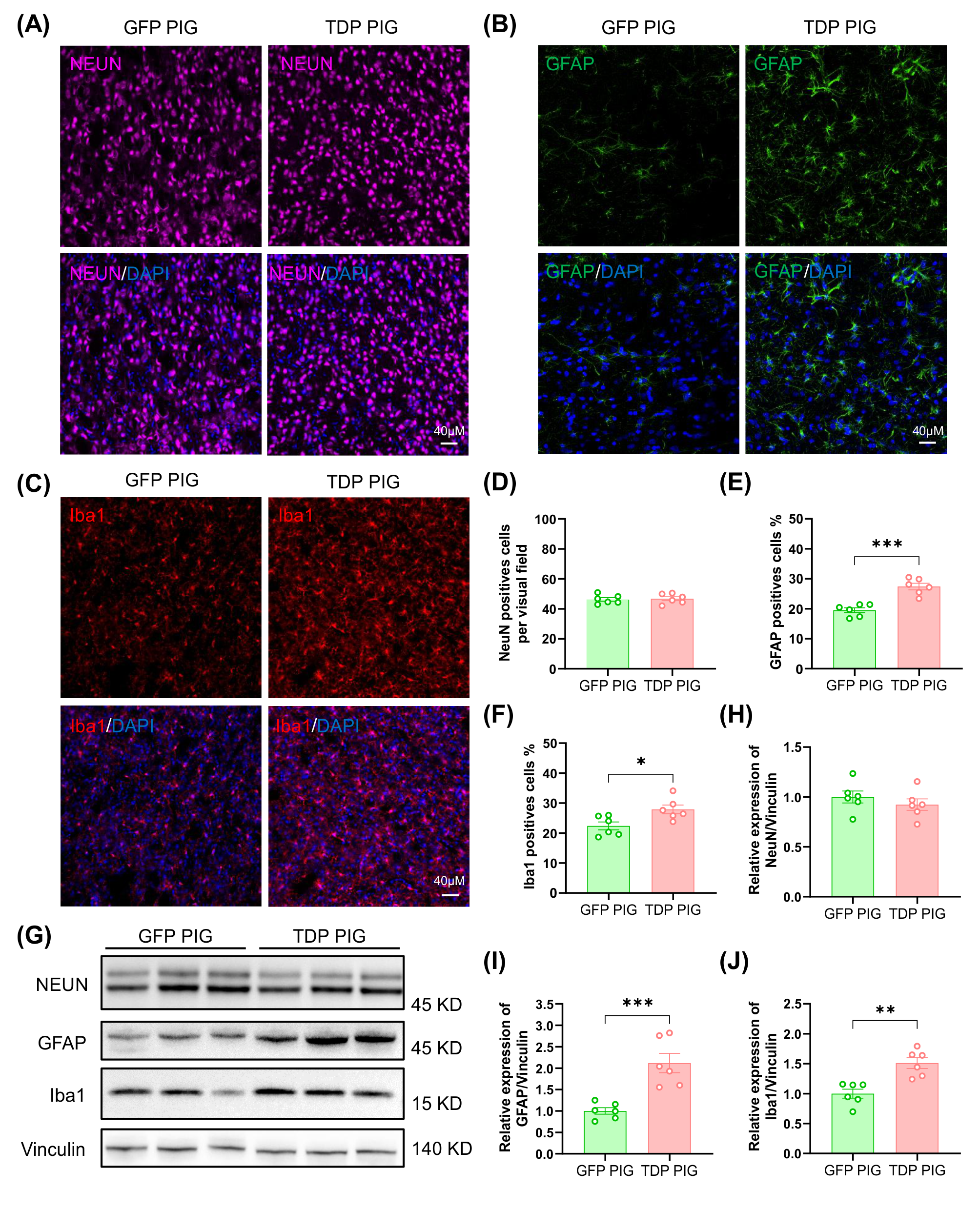

Supplement: Supplementary file 2 — Figure S2.tif [file MCO2-6-e70330-s004.tif]

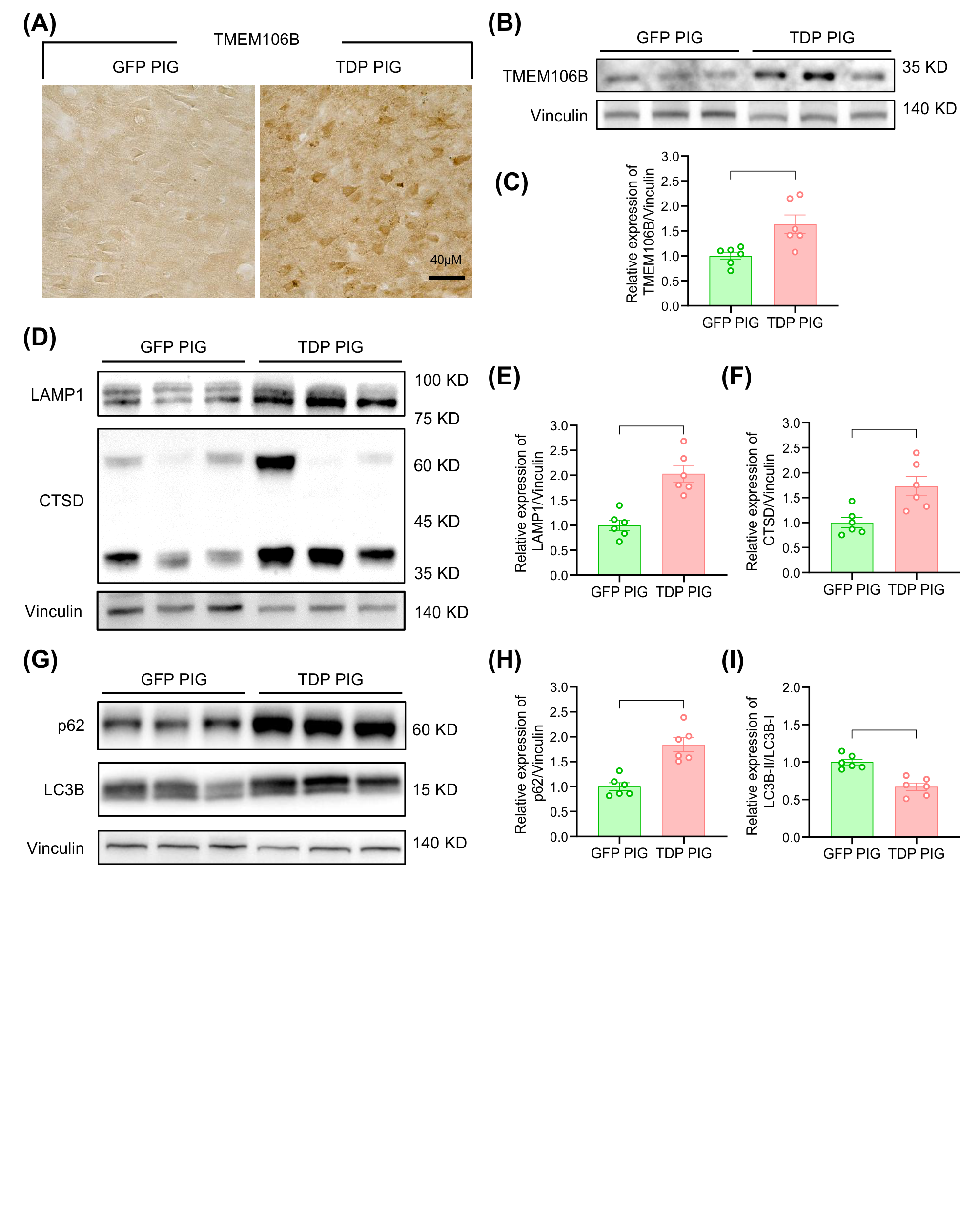

Supplement: Supplementary file 3 — Figure S3.tif [file MCO2-6-e70330-s005.tif]
